# Important genetic checkpoints for insulin resistance in salt-sensitive (S) Dahl rats

**DOI:** 10.1186/1475-2840-7-19

**Published:** 2008-06-21

**Authors:** Marlene F Shehata

**Affiliations:** 1 Department of Cellular and Molecular Medicine, University of Ottawa Heart Institute, K1Y 4W7, Ottawa, ON, Canada

## Abstract

Despite the marked advances in research on insulin resistance (IR) in humans and animal models of insulin resistance, the mechanisms underlying high salt-induced insulin resistance remain unclear. Insulin resistance is a multifactorial disease with both genetic and environmental factors (such as high salt) involved in its pathogenesis. High salt triggers insulin resistance in genetically susceptible patients and animal models of insulin resistance. One of the mechanisms by which high salt might precipitate insulin resistance is through its ability to enhance an oxidative stress-induced inflammatory response that disrupts the insulin signaling pathway. The aim of this hypothesis is to discuss two complementary approaches to find out how high salt might interact with genetic defects along the insulin signaling and inflammatory pathways to predispose to insulin resistance in a genetically susceptible model of insulin resistance. The first approach will consist of examining variations in genes involved in the insulin signaling pathway in the Dahl S rat (an animal model of insulin resistance and salt-sensitivity) and the Dahl R rat (an animal model of insulin sensitivity and salt-resistance), and the putative cellular mechanisms responsible for the development of insulin resistance. The second approach will consist of studying the over-expressed genes along the inflammatory pathway whose respective activation might be predictive of high salt-induced insulin resistance in Dahl S rats.

Variations in genes encoding the insulin receptor substrates -1 and/or -2 (IRS-1, -2) and/or genes encoding the glucose transporter (GLUTs) proteins have been found in patients with insulin resistance. To better understand the combined contribution of excessive salt and genetic defects to the etiology of the disease, it is essential to investigate the following question:

Question 1: Do variations in genes encoding the IRS -1 and -2 and/or genes encoding the GLUTs proteins predict high salt-induced insulin resistance in Dahl S rats?

A significant amount of evidence suggested that salt-induced oxidative stress might predict an inflammatory response that upregulates mediators of inflammation such as the nuclear factor- kappa B (NF-kappa B), the tumor necrosis factor-alpha (TNF-α) and the c-Jun Terminal Kinase (JNK). These inflammatory mediators disrupt the insulin signaling pathway and predispose to insulin resistance. Therefore, the following question will be thoroughly investigated:

Question 2: Do variations in genes encoding the NF-kappa B, the TNF-α and the JNK, independently or in synergy, predict an enhanced inflammatory response and subsequent insulin resistance in Dahl S rats in excessive salt environment?

Finally, to better understand the combined role of these variations on glucose metabolism, the following question will be addressed:

Question 3: What are the functional consequences of gene variations on the rate of glucose delivery, the rate of glucose transport and the rate of glucose phosphorylation in Dahl S rats?

The general hypothesis is that "high-salt diet in combination with defects in candidate genes along the insulin signaling and inflammatory pathways predicts susceptibility to high salt-induced insulin resistance in Dahl S rats".

## Background

### Insulin Resistance

Insulin resistance is defined as an impaired responsiveness to both endogenous and exogenous insulin resulting in high blood glucose levels and compensatory hyperinsulinemia [1]. Insulin resistance predicts a constellation of risk factors that increase the risk of cardiovascular diseases. These include high blood pressure, obesity, type 2 diabetes, elevated triglycerides, and lowered high density lipoprotein cholesterol (HDL-C) [2,3]. Statistics have shown that over 30% of Canadian adults have insulin resistance [4] and that about 50% of salt-sensitive subjects are insulin resistant [3]. The prevalence of the disease is expected to increase dramatically over the coming decades causing an enormous economic burden in both the developing and developed worlds.

Insulin resistance has major genetic and environmental factors (such as dietary salt) that are involved in its pathogenesis [1]. Despite the intense investigations into the mechanisms of insulin resistance, the contribution of high-salt to insulin resistance is not yet clear and the genes responsible for the development of high salt-induced insulin resistance remain in dispute. Because insulin resistance phenotype results from the cumulative effects of several contributing genetic alterations, I chose to focus on the genetic differences in candidate genes along the insulin signaling and inflammatory pathways.

Variations in major genes encoding the insulin receptor substrates -1 and -2 (IRS-1 and -2) and the glucose transporter (GLUTs) proteins correlate with insulin resistance in humans and animal models of insulin resistance. These variations act by different mechanisms to disrupt the insulin signaling pathway leading to defects in glucose transport and glucose metabolism.

Multiple lines of evidence suggest a putative role of salt-induced oxidative stress in the etiology of insulin resistance [5]. Salt-induced oxidative stress triggers a powerful inflammatory response that disrupts the insulin signaling pathway and might possibly explain insulin resistance in genetically susceptible populations [6-8]. Owing to the fact that high salt-fed Dahl S rats have augmented salt-induced oxidative stress in most tissues [9-12], and that oxidative stress generates mediators of inflammation such as NF-kappa B, TNF-α and JNK, known to disrupt the insulin signaling pathway, I chose to review the putative genetic variations in the markedly over-expressed genes along the inflammatory pathway (NF-kappa B, TNF-α and JNK) that might be predictive of insulin resistance in Dahl S rat model. Because the promoter regions of some of the candidate genes proposed in the current study might possibly harbor sodium-binding sequences [13,14], any sequence variations in these putative sodium response elements might predict protection against insulin resistance in Dahl R rats in excessive salt environments. The long-term goal is to establish a subset of single nucleotide polymorphisms (SNPs) that are predictive of insulin resistance in excessive salt environments.

### Why Dahl rats?

Dahl inbred rat strains are classified into salt-sensitive (S) and salt-resistant (R) based on their blood pressure responses to high salt diet. Dahl S and R rats share 80% of their genetic background [15]. Contrary to Dahl R, Dahl S rats represent a robust model of insulin-resistance syndrome on account of their insulin resistance, hypertriglyceridaemia, abdominal obesity and salt-sensitive hypertension that is exacerbated on dietary sodium intake [16-19]. Dahl S rats alone had significant insulin resistance, hyperinsulinemia, elevated systolic and diastolic blood pressures on high salt diet (8% NaCl) for 4 weeks *versus *normal salt diet [19,20]. Whole body insulin resistance in Dahl S rats was evidenced by a decrease in glucose utilization during the hyperinsulinemia-euglycemic clamp analysis while peripheral insulin resistance in Dahl S rats was evidenced by a decrease in insulin-stimulated 2 deoxy-glucose uptake by adipocytes and skeletal muscles obtained from high-salt-fed Dahl S *versus *Dahl R rats. At the molecular level, tyrosyl phosphorylation of the insulin receptor, insulin receptor substrates and PI3K, as well as serine phosphorylation of Akt were all enhanced in Dahl S *versus *R rats [19].

Insulin resistance in Dahl S rats does not seem to develop as a result of an alteration in the insulin receptor number, affinity, binding parameters, mRNA levels or tissue distribution in liver, muscle and kidney tissues. These parameters were all comparable in Dahl S and R rats on high or low salt chow [20]. Moreover, the muscle and adipose tissue glucose transporter protein 4 (GLUT 4) expression was comparable in soleus muscle and adipose tissue of Dahl S and R rats on low and high salt diet [19]. Insulin resistance in Dahl S rats is worsened on high salt diet, and high salt diet enhances oxidative stress-induced inflammatory mediators such as TNF-α, JNK, and NF-kappa B in Dahl S rats, therefore, high salt-induced oxidative stress might predict a powerful inflammatory response in Dahl S *versus *R rats. This powerful inflammatory response might be caused by genetic differences that enhances the expression of inflammatory mediators and predispose Dahl S rats to insulin resistance.

**Figure 1:**
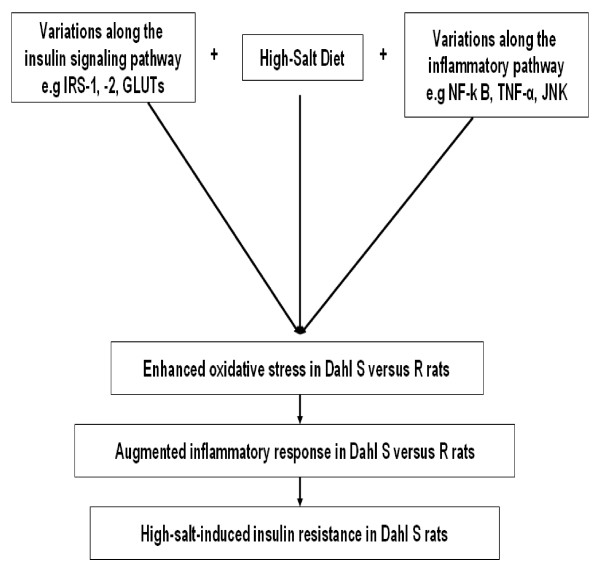
**Mechanism of High Salt-Induced Insulin Resistance in Dahl S rats.** Our proposed model of high salt-induced insulin resistance in Dahl S rats depends on the combined contributions of high salt and genetic defects along the insulin signaling and inflammatory pathways, which in turn predispose Dahl S rats to an augmented inflammatory response and overt insulin resistance.

In conclusion, the lack of change in the insulin receptor number in combination with multiple changes in post receptor signaling suggests a post receptor defect. The decreased sensitivity to insulin in Dahl S rats may arise from a common variant (s) in genes along the insulin signaling and/or inflammatory pathways that triggers a powerful inflammatory response and enhance overt insulin resistance in Dahl S rats in excessive salt environment (Figure 1).

### Factors along the insulin signaling pathway with implications in insulin resistance

#### Insulin receptor substrates -1 and -2 (IRS-1 and IRS-2)

**Figure 2:**
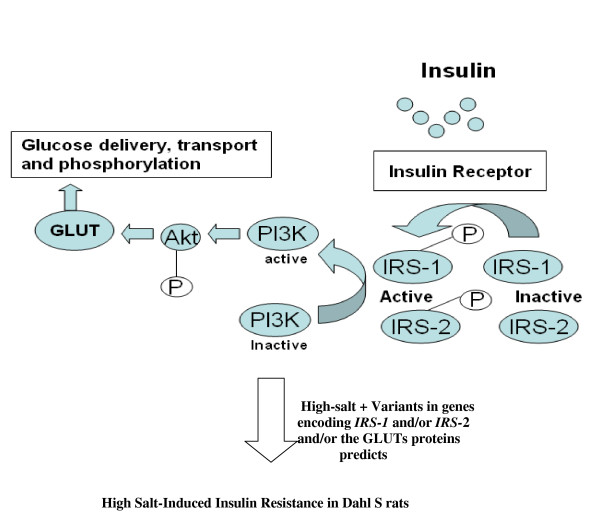
**Illustration of the Insulin Pathway and its Potential Contribution to High Salt-Induced Insulin Resistance in Dahl S rats**. When insulin activates the insulin receptor in vivo, tyrosine phosphorylation of the IRS-1 and IRS-2 activates phosphoinositide 3 kinase (PI3K) enzyme, whose activation in turn stimulates the serine phosphorylation of Akt (Protein kinase B) [108]. The latter enzyme (PKB) stimulates glucose transport in muscle and adipose tissue through the stimulated translocation of glucose transporter GLUT4 isoform [109]. PKB also stimulates glycogen synthesis in the liver and muscle, and stimulates lipogenesis in adipose tissue. Existence of variants in genes encoding IRS-1 and/or-2 and glucose transporter proteins might impair insulin signaling by suppressing the IRS-1 and/or -2 mRNA levels, protein levels and activity or enhancing serine phosphorylation of IRS-1 and -2 resulting in high salt-induced insulin resistance in Dahl S rats.

Insulin effects are predominant in skeletal muscles, liver, kidney, fat and brain causing increased renal sodium retention, modulation of transmembrane cation transport, induction of growth promoting effects of vascular smooth muscle cells and vascular hyperreactivity [21]. Insulin actions are initiated when insulin binds to a high-affinity heterotetrameric transmembrane protein receptor that is present in all mammalian cells [22]. This is followed by activation of second messengers, also called docking proteins, such as the insulin receptor substrates-1, -2, -3 and -4, (IRS-1, -2, -3 and -4) [23-26] via a series of phosphorylation-dephosphorylation reactions that result in stimulation of glucose metabolism [27] (Figure 2). While IRS-3 and -4 play a role in cell growth and differentiation, IRS-1 and -2 play an important role in glucose metabolism and represent attractive candidate genes to study in insulin resistance (Tables 1 and 2).

**Table 1:** Chromosomal location, genomic and protein sizes of IRS-1 and IRS-2 in rats

**Gene**	**Location**	**Size**	**Size of Coding region**	**Justification**
Insulin receptor substrate 1 (IRS-1)	chr9:81585251–81638071	5365 bases	3708 bases	Low cellular IRS 1 gene and protein expression predict insulin resistance and NIDDM. Impairment of insulin-induced glucose uptake by skeletal muscles from high-salt-fed Dahl S rats was neither due to changes in the insulin receptor number, mRNA or binding affinity, nor due to diminished expression of GLUT4 protein. Impairment in the insulin signaling pathway might possibly be downstream of the insulin receptor and upstream of GLUT4, possibly in the *IRS-1 *and/or *IRS-2 *signaling molecules.
Insulin receptor substrate 2 (IRS-2)	chr16:18878139–18889441	24234 bases	3963 bases	Ubiquitin-mediated degradation of IRS1 and IRS2 promotes insulin resistance. IRS2 dysfunction is critical in the development of type 2 diabetes. Insulin was not able to suppress gluconeogenic gene expression in primary hepatocytes lacking IRS-2, but when IRS-2 signaling was reconstituted, these cells recovered this response to insulin.

Summary of *IRS-1 *and *-2 *genes demonstrating their chromosomal location, respective genomic and coding sequence sizes, and known effects of their dysregulation.

**Table 2:** Previously identified variant (s) in IRS-1 gene in rats

**Gene**	**Strain**	**Type**	**Residue**	**SNP**	**Amino Acid**
**IRS-1**	Sprague Dawley Ref: Brown Norway	Non-synonymous	1160	ENSRNOSNP2552231	T/C

Table showing previously reported IRS-1 variant in Sprague Dawley rats compared to Brown Norway rats. The IRS-1 non-synonymous variant caused an amino acid change from Cysteine to Tyrosine. Functional assessment of such variant is yet to be determined [133].

**Figure 3:**
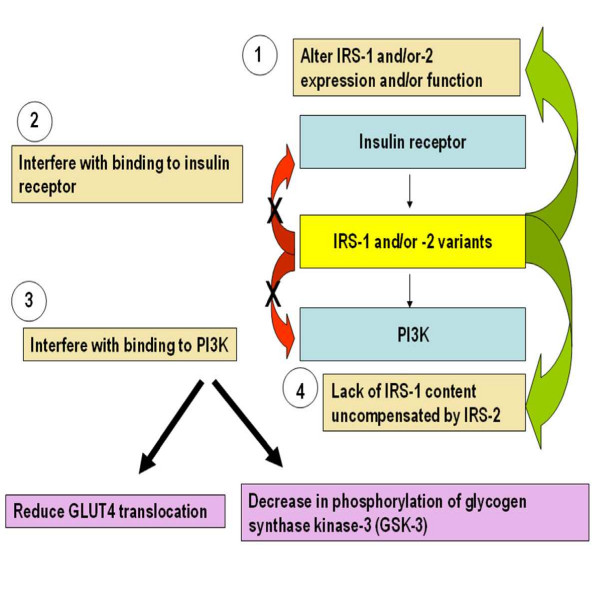
**Contribution of IRS-1 and/or-2 variants to Insulin Resistance**. Mechanisms by which IRS-1 and/or -2 variants contribute to insulin resistance include: 1) altering IRS-1 and/or-2 expression and function, 2) reduced IRS-1 and/or -2 binding to the insulin receptor, 3) a defect in binding of IRS-1 and/or -2 variant (s) to the p85 regulatory subunit of the PI3-kinase and a decrease in PI3-kinase activity. The latter leads to either a decreased GLUT4 translocation to the plasma membrane, further reducing glucose transport and glycogen synthesis, or a significant IRS-1 and/or -2 -induced decrease in phosphorylation of glycogen synthase kinase-3 (GSK-3), an enzyme that is important in glycogen synthesis, thus causing reduced glycogen synthesis, 4) reduced IRS-1 content that is not compensated by a constitutive increase in the IRS-2 protein content. This result in a reduced insulin-stimulated PI3-kinase activity and a significant decrease in Akt phosphorylation and activity.

In humans, associations between insulin resistance and common variants in *IRS-1 *and *-2 *have been reported in several populations [25,28-36], including obese Caucasian children, adults, Asian Indians, Mexicans and Europeans. Mechanisms underlying the contribution of IRS-1 and/or -2 variants to insulin resistance include [37] (Figure 3): i) altering IRS-1 and/or-2 expression and function, ii) reduced IRS-1 and/or -2 binding to the insulin receptor, iii) a defect in binding of IRS-1 and/or -2 variant (s) to the p85 regulatory subunit of the PI3-kinase and a decrease in PI3-kinase activity. This in turn leads to either a decreased GLUT4 translocation to the plasma membrane, further reducing glucose transport and glycogen synthesis, or a significant IRS-1 and/or -2 -induced decrease in phosphorylation of glycogen synthase kinase-3 (GSK-3), an enzyme that is important in glycogen synthesis, thus causing reduced glycogen synthesis, iv) reduced IRS-1 content that is not compensated by a constitutive increase in the IRS-2 protein content. This result in a reduced insulin-stimulated PI3-kinase activity and a significant decrease in Akt phosphorylation and activity. Overall, IRS-1 and/or 2 variants seem to impair the ability of insulin to activate the IRS/PI3-kinase/Akt/GSK-3 signaling pathway leading to defects in glucose transport, glucose transporters translocation and glycogen synthesis.

**Figure 4:**
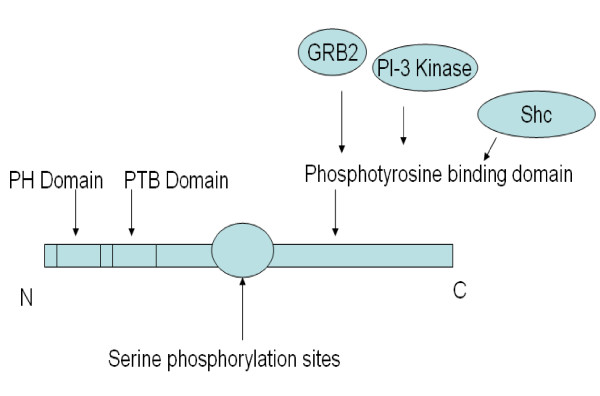
**Structural Determinants of Insulin Receptor Substrates**. IRS-1 and -2 are considerably similar in their general architecture [23,24,38]. They are composed of an NH2-terminal, pleckstrin homology (PH) domain that binds to membrane phospholipids, a phosphotyrosine binding (PTB) domain located just upstream the PH domain and is involved in the recognition of the asparagines-proline-glutamic acid-phosphotyrosine (NPEpY) sequence located in the juxtamembrane region of the insulin receptor β subunit, phosphoserine binding domain (PSB), and a less conserved COOH-terminal portion with multiple tyrosine phosphorylation motifs that can bind to specific SH2 domain-containing proteins.

**Figure 5:**
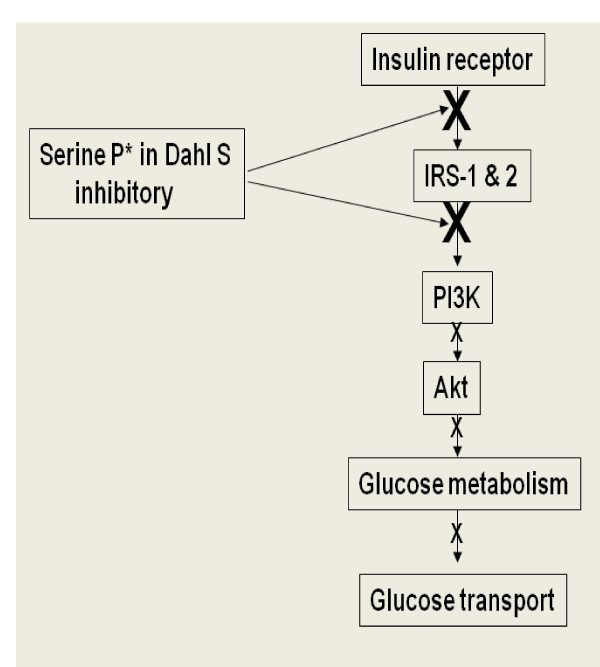
**Serine phosphorylation in Dahl S rats precipitates insulin resistance**. Dahl S rats are postulated in the current proposal to have increased serine phosphorylation of insulin receptor substrate-1 and/or 2 (IRS-1 and/or-2) on critical sites such as Ser 312, 616, 307, and 323. This in turn reduces their ability to bind and activate either the insulin receptor or the phosphoinositol 3-kinase, resulting in reduced insulin-stimulated glucose transport activity and other events downstream of PI3K.

IRS-1 and -2 proteins are subjected to phosphorylation on either tyrosine or serine residues (Figure 4 for structural organization). IRS-1 and -2 phosphorylation on tyrosine residues activates IRS proteins to bind to signaling molecules containing SH2 domains, including PI3K [24,38]. Unlike tyrosyl phosphorylation, serine phosphorylation of IRS proteins attenuated insulin signaling and might potentially explain an additional mechanism of insulin resistance in rodents [39] (Figure 5). Owing to the fact that IRS proteins have three times the number of serine residues compared to the number of tyrosine residues, the significance of serine phosphorylation has been significantly highlighted. Serine phosphorylation of IRS-1 and IRS-2 is the main mechanism of suppressing insulin signaling [40,41] in the following ways [40-42]: 1) They can induce dissociation of IRS proteins from the insulin receptor, 2) hinder tyrosine phosphorylation sites and release IRS proteins from intracellular complexes that maintain them in close proximity to the receptor, 3) induce IRS protein degradation, or 4) turn IRS proteins into inhibitors of the insulin receptor kinase. Differential serine phosphorylation on IRS-1 and/or-2 has not been studied in Dahl S *versus *R rats. Additionally, serine phosphorylation of the insulin receptor and IRS-1 and -2 [43] is enhanced by chronic hyperinsulinemia that negatively modulate insulin signaling via activation of TNF-α [43]. Therefore, chronic hyperinsulinemia in high-salt-fed-Dahl S rats [16] might as well enhance serine phosphorylation on IRS-1 and/or IRS-2 and explain insulin resistance in Dahl S rats.

#### Glucose Transporters

The ability to transport glucose across plasma membranes is mediated by members of the glucose transporter proteins (Table 3). Two families of glucose transporter have been identified: i) GLUTs: facilitated-diffusion glucose transporter family also known as 'uniporters,' (GLUT1 through GLUT4), ii) SGLTs: sodium-dependent glucose transporter family, also known as 'cotransporters' or 'symporters', transport glucose against its concentration gradient.

**Table 3:** Chromosomal location, tissue distribution and transcript information of genes encoding glucose transporters

**Gene Symbol**	**Gene name**	**Chromosomal Location**	**Tissue Distribution**	**Function**	**Transcript Information**
Slc2a1	facilitated glucose transporter member 1 (Glucose transporter type 1) (GLUT-1)	chr5:139690804–139719021	All tissues (abundant in brain and erythrocytes)	Basal uptake	**Exons: **10 **Transcript length: **2,365 bps **Protein length: **492 residues
Slc2a2	solute carrier family 2, facilitated glucose transporter member 2 (Glucose transporter type 2) (GLUT-2)	chr2:116036427–116065870	Liver, pancreatic islet cells, retina	Glucose sensing	**Exons: **12 **Transcript length: **2,578 bps **Protein length: **524 residues
Slc2a3	solute carrier family 2, facilitated glucose transporter member 3 (Glucose transporter type 3) (GLUT-3)	chr4:159210019–159221248	All tissues specially the brain	Supplements GLUT1 in tissues with high energy demand	**Exons: **9 **Transcript length: **1,467 bps **Protein length: **488 residues
Slc2a4	solute carrier family 2, facilitated glucose transporter member 4 (Glucose transporter type 4) (GLUT-4).	chr10:56,786,705–56,792,209.	Muscle, fat, heart	Insulin responsive	**Exons: **11 **Transcript length: **2,506 bps **Protein length: **509 residues
Slc2a8	solute carrier family 2, facilitated glucose transporter member 8 (Glucose transporter type 8) (GLUT-8)	chr3:11962578–11972099	hippocampal neurons, testis, whole brain		**Exons: **10 **Transcript length: **2,087 bps **Protein length: **478 residues
Naglt1	Sodium dependant glucose transporter 1	chr20:44,147,331–44,176,308.	In all cells		**Exons: **4 **Transcript length: **1,455 bps **Protein length: **484 residues
Slc37a4	solute carrier family 37 (glucose-6-phosphate transporter), member 4	chr 8: 47,363,896–47,369,981.	Liver, Adipocytes		**Exons: **9 **Transcript length: **2,080 bps **Protein length: **429 residues

In humans, 23, 38, 2, 1 and 41 mutations have been reported in GLUT 1, GLUT 2, GLUT 4, GLUT 10, and the SGLT 1, respectively. These mutations have been associated with glucose transporters deficiency, lower serum insulin levels, glucose malabsorption and NIDDM making them attractive molecular targets for insulin resistance and glucose abnormalities [44] (For review, the Human Gene Mutation Database).

In rats, at least 7 genes are involved in glucose transport. They are denoted by Slc2a1, Slc2a2, Slc2a3, Slc2a4, Slc2a8, Slc37a4, Naglt1 (Table 3). Despite the reported variations in some of the above genes in rats (Table 4), no further functional studies have been reported so far. Using the TRANSFAC ^® ^computer program [45], sodium-binding sequences [13,14] were identified in the promoters of Slc2a1, Slc2a2, and Slc2a4. As such, association between high salt and insulin resistance might be through the glucose transporter genes.

**Table 4:** Previously reported variant (s) in genes encoding glucose transporter proteins

**Gene**	**Strain**	**Type**	**Residue**	**SNP**	**Amino Acid**
**Slc2a1**	Sprague Dawley Ref: Brown Norway	Synonymous	383	ENSRNOSNP1925604	C/T
**Slc2a2**	Sprague Dawley Ref: Brown Norway	Non-synonymous	475	ENSRNOSNP1165255	A/G
	Sprague Dawley Ref: Brown Norway	Non-synonymous	512	ENSRNOSNP1165256	G/A
	Sprague Dawley Ref: Brown Norway	Synonymous	520	ENSRNOSNP1165257	T/A
**Slc2a3**	Sprague Dawley Ref: Brown Norway	Non-synonymous	50	rs8172435	G/C
	Sprague Dawley Ref: Brown Norway	Synonymous	147	rs8160316	A/C
	Sprague Dawley Ref: Brown Norway	Non-synonymous	442	rs8172437	A/G
	Sprague Dawley Ref: Brown Norway	Non-synonymous	465	rs8172438	C/G
**Naglt1**	Sprague Dawley Ref: Brown Norway	Non-synonymous	166	ENSRNOSNP1513056	C/A

Previously reported variants in the glucose transporters in the Sprague Dawley rat. The reference strain was the Brown Norway rat [133].

#### Factors along the inflammatory pathways

Inflammation causes a profound change in gene expression of a large number of inflammatory proteins such as tumor necrosis factor-alpha (TNF-α), c-Jun Terminal Kinase (JNK) and nuclear factor-kappa B, (NF-kappa B) which increases oxygen consumption and produce many reactive oxygen species (ROS). ROS might as well be triggered by high salt [46]. ROS interfere with normal insulin signaling and precipitate insulin resistance via a number of mechanisms including: i) disruption of insulin-induced IRS-1 and PI3-kinase phosphorylation and cellular distribution, ii) reduction in GLUT4 expression levels, or iii) reduction in GLUT4 translocation to the plasma membrane [47-50]. High salt-fed Dahl S rats had augmented NADPH oxidase activity (oxidative stress inducer) in cardiac [10], renal [9,11,12], mesenteric microvascular [9] and brain tissues [51]. Even plasma levels of hydrogen peroxide (oxidative-stress inducer) in high-salt-fed Dahl S rats were significantly elevated [9], and the renal antioxidant, superoxide dismutase levels were diminished [9]. As such, high salt-induced oxidative stress might predict a powerful inflammatory response in Dahl S *versus *R rats because of genetic differences along the inflammatory pathway that predispose them to perturbations in the expression of inflammatory genes.

Table 5 includes a list of inflammatory genes (pro- and anti-inflammatory factors) with potential implications in insulin resistance. While there are a plethora of genes associated with inflammation and insulin resistance, I will focus on a select few TNF-α, JNK, NF-kappa B which based on the literature are plausible candidates. TNF-α and JNK are potential mediators of insulin resistance and are significantly upregulated in high-salt-fed-Dahl S rats [52,53]. TNF-α and JNK contribute to insulin resistance by: i) promoting serine phosphorylation of IRS-1 and -2, ii) formation of a stable complex with IRS-1 and/or IRS-2, iii) impairing the ability of IRS-1 and -2 to associate with the insulin receptor and inhibiting insulin-stimulated tyrosine phosphorylation [54-57].

**Table 5:** List of Major Candidate Genes Involved in the Inflammation Pathway

**Gene Symbol**	**Gene Name**	**Chromosomal Location**	**Transcript information**	**Justification**
**TNF-α**	tumor necrosis factor receptor superfamily	chr3:156092602–156107426	**Exons: **9 **Transcript length: **1,247 bps **Protein length: **289 residues	TNF-α, a potential mediator of insulin resistance, promotes serine phosphorylation of IRS-1 and -2, impairs the ability of IRS-1 and -2 to associate with the insulin receptor and inhibits insulin-stimulated tyrosine phosphorylation [54–56]. TNF-α is upregulated in Dahl S rats [52].
**NF-kappa-B-activating protein**	Nuclear factor kappa-B-activating protein	chr X: 7,762,299–7,781,765.	**Exons: **9 **Transcript length: **1,248 bps **Protein length: **415 residues	Renal NF-{kappa}B is significantly upregulated in high-salt-fed Dahl S rats [58].
**NF-IKBKB**	Nuclear factor kappa-B kinase subunit beta	chr16: 73,805,082–73,858,088.	**Exons: **21 **Transcript length: **3,022 bps **Protein length: **757 residues	Inhibition of IKBKB with salicylates or through targeted gene disruption causes a dramatic improvement of insulin sensitivity in animal models of insulin resistance such as ob/ob mice and obese Zucker fatty rats [59,60].
**IL1β**	Interleukin-1β receptor accessory protein precursor	chr11: 76,092,840–76,222,495.	**Exons: **11 **Transcript length: **1,862 bps **Protein length: **570 residues	IL1β activates jnk which is upregulated in high-salt-fed Dahl S rats [57].
**IL17d**	interleukin 17D	chr 15: 36,566,307–36,583,168.	**Exons: **9 **Transcript length: **621 bps **Protein length: **206 residues	IL-17 D, a proinflammatory cytokine that enhances T cell priming and stimulates the production of proinflammatory molecules such as IL-1, IL-6, TNF-alpha, NOS-2, and chemokines resulting in inflammation.
**IL10**	Interleukin-10 precursor (IL-10) (Cytokine synthesis inhibitory factor) (CSIF).	chr 13: 43.95m	**Exons: **5 **Transcript length: **1,289 bps **Protein length: **178 residues	IL-10, also known as human cytokine synthesis inhibitory factor (CSIF), is an anti-inflammatory cytokine. This cytokine can block NF-kappa B activity, and is involved in the regulation of the JAK-STAT signaling pathway. It is capable of inhibiting synthesis of pro-inflammatory cytokines like Interferon-gamma, IL-2, IL-3, TNFα and GM-CSF made by cells such as macrophages and the Type 1 T helper cells.
**Crp**	C-reactive protein precursor	chr 13: 88,674,743–88,715,585.	**Exons: **2 **Transcript length: **1,655 bps **Protein length: **230 residues	Insulin resistance and C-reactive protein (CRP) levels are strongly correlated in adults [110].
**Ratsg2**	Selenoprotein S (VCP-interacting membrane protein) (Sg2).	chr 1: 120,509,128–120,518,322.	**Exons: **6 **Transcript length: **573 bps **Protein length: **190 residues	In humans, polymorphisms in the encoded plama membrane selenoprotein (SEPS1, or SELS gene) correlate to diabetes mellitus and coronary heart diseases. The selenoprotein regulate red-ox balance and clear cells of misfolded proteins. Gene polymorphisms result in accumulation of these proteins even higher under cell stress. Carriers have higher IL1, -6, -10, and TNF [111].
**Ptpn22_predicted**	protein tyrosine phosphatase, non-receptor type 22 (lymphoid) (predicted)	chr 2: 199,083,234–199,132,761	**Exons: **22 **Transcript length: **2,476 bps **Protein length: **803 residues	Tyrosine phosphatase gene (PTPN22) prevents spontaneous T-cell activation. In humans, mutations (C1858T, R620W) was associated with type 1 diabetes [112–114].
**Crhr1**	Corticotropin-releasing factor receptor 1 precursor (CRF-R) (CRF1) (Corticotropin-releasing hormone receptor 1) (CRH-R 1).	chr 10: 93.31 m	**Exons: **12 **Transcript length: **1,218 bps **Protein length: **405 residues	Crhr1 is required for a normal chromaffin cell structure and function and deletion of this gene is associated with a significant impairment of epinephrine release.
**IL6**	Interleukin-6 precursor	chr 4: 456,799–461,376.	**Exons: **6 **Transcript length: **1,042 bps **Protein n length: **210 residues	Impaired glucose tolerance is associated with increased serum concentrations of interleukin 6 [115].
**IL15**	Interleukin-15 precursor.	chr 19: 27,482,376–27,499,255.	**Exons: **6 **Transcript length: **768 bps **Protein length: **161 residues	IL-15 increases insulin sensitivity therefore increasing glucose transport and utilization in muscles [116].
**IL18**	interleukin 18	chr 9: 39,676,026–39,698,748.	**Exons: **10 **Transcript length: **1,884 bps **Protein length: **604 residues	Elevated plasma interleukin-18 is a marker of insulin-resistance in type 2 diabetic and non-diabetic humans [117,118].
**Map2k7**	Dual specificity mitogen-activated protein kinase kinase 7	chr 12: 1,543,467–1,552,353.	**Exons: **13 **Transcript length: **1,407 bps **Protein length: **468 residues	MAP2K7 selectively activates the JNKs which suppresses insulin signaling [57].
**Mapk6**	Mitogen-activated protein kinase 6 or (Extracellular signal-regulated kinase 3) (ERK-3) (p55-MAPK).	chr 8: 80,212,726–80,236,362	**Exons: **6 **Transcript length: **4,180 bps **Protein length: **720 residues	ERK3 associates with MAP2 and is involved in glucose-induced insulin secretion [119].
**Map4k4**	Mitogen-activated protein kinase 4-isoform4	chr 9: 39,070,845–39,211,446.	**Exons: **34 **Transcript length: **4,401 bps **Translation length: **1,232 residues	Map4k4 gene silencing in human skeletal muscle prevents tumor necrosis factor-alpha-induced insulin resistance [120].
**Map2k1**	Dual specificity mitogen-activated protein kinase kinase 1 or (MAP kinase kinase 1) (MAPKK 1) (ERK activator kinase 1) (MAPK/ERK kinase 1) (MEK1).	chr 8: 68,379,077–68,451,583.	**Exons: **11 **Transcript length: **2,120 bps **Protein length: **393 residues	MAP2K1 restored insulin action on glucose uptake by cells [120,121].
**Jnk**	C-Jun amino terminal kinase	chr 3: 76.78 m	**Exons: **12 **Transcript length: **2,992 bps **Protein length: **699 residues	JnK is activated by TNF-α and IL-β. Jnk forms a stable complex with IRS-1 and phosphorylates Ser307 that inhibits insulin stimulated tyrosine phosphorylation of IRS-1 [57].
**Cx3cr1**	CX3C chemokine receptor 1	chr 8: 125.03 m	**Exons: **2 **Transcript length: **1,326 bps **Protein length: **354 residues	Modulators of CX3CR1 can be used to treat diabetes, as well as diagnose diabetes by measuring the levels of CX3CR1 in a patient (US patents 2006).
**Ccr3**	C-C chemokine receptor type 3	chr 8: 128.76 m	**Exons: **2 **Transcript length: **1,315 bps **Protein length: **359 residues	CCL3was reported to be increased in obese mice and to contribute to insulin resistance and macrophage recruitment [122].
**Ccr2**	C-C chemokine receptor type 2	chr 8: 128.89 m	**Exons: **1 **Transcript length: **1,122 bps **Protein length: **373 residues	CCR2 influences the development of obesity and associated adipose tissue inflammation and systemic insulin resistance [123].
**Ccr5**	C-C chemokine receptor type 5	chr 8: 128.91 m	**Exons: **2 **Transcript length: **2,495 bps **Protein length: **354 residues	CCR5 polymorphisms in children with insulin-dependent diabetes mellitus [124].
**Nox3**	NADPH oxidase 3	chr 1: 38.64 m	**Exons: **14 **Transcript length: **1,761 bps **Protein length: **586 residues	NOX3, a ROS generating NADPH oxidase, plays an integral role in insulin-induced signal transmission [125].
**Nox4**	NADPH oxidase 4	chr 1: 143.42 m	**Exons: **18 **Transcript length: **2,176 bps **Protein length: **578 residues	The NAD(P)H Oxidase Homolog Nox4 Modulates Insulin-Stimulated Generation of H2O2 and Plays an Integral Role in Insulin Signal Transduction [126].
**Ptgs2**	Prostaglandin G/H synthase 2 precursor (Cyclooxygenase-2) (COX-2) (Prostaglandin H2 synthase 2) (PGH synthase 2) (PGHS-2) (PHS II).	chr 13: 64,427,282–64,432,982.	**Exons: **10 **Transcript length: **1,825 bps **Protein length: **604 residues	PTGS2 generates prostaglandins, which negatively modulate glucose-stimulated insulin secretion, and functions as a mediator of the inflammatory response [127].
**Alox5**	Arachidonate 5-lipoxygenase (5-lipoxygenase) (5-LO)	chr 4: 152.61 m	**Exons: **14 **Transcript length: **2,450 bps **Protein length: **674 residues	The epidemiologic data suggest that subjects with two variant alleles will have greater ALOX5 gene expression, greater production of arachidonic acid-derived leukotrienes and a more "proinflammatory phenotype than subjects with two common alleles.
**Alox5ap**	Arachidonate 5-lipoxygenase-activating protein (FLAP) (MK-886-binding protein).	chr 12: 6.25 m	**Exons: **5 **Transcript length: **937 bps **Protein length: **161 residues	ALOX5AP expression, but not gene haplotypes, is associated with obesity and insulin resistance [128].
**Nos3**	Nitric-oxide synthase, endothelial (NOSIII) (Endothelial NOS) (eNOS)	chr 4: 6.16 m	**Exons: **26 **Transcript length: **3,953 bps **Protein length: **1,202 residues	The (-)786T-C mutation of the eNOS gene is associated with insulin resistance in both Japanese non-diabetic subjects and Type II diabetic patients [129]
**Nos2**	Nitric oxide synthase, inducible (NOS type II) (Inducible NO synthase) (Inducible NOS) (iNOS)	chr 10: 65.04 m	**Exons: **27 **Transcript length: **4,106 bps **Protein length: **1,147 residues	obese *Nos2 *^-/- ^mice exhibited improved glucose tolerance, normal insulin sensitivity *in vivo *and normal insulin-stimulated glucose uptake in muscles [130].
**Cpr**	NADPH-cytochrome P450 reductase (CPR) (P450R).	chr 12: 22.08 m	**Exons: **16 **Transcript length: **2,438 bps **Protein length: **678 residues	NADPH-cytochrome P450 reductase (CPR) plays a role in type II diabetes [131]
**Pla2g1b**	Phospholipase A2 precursor	chr 12: 42.41 m	**Exons: **4 **Transcript length: **543 bps **Protein length: **146 residues	Mice with targeted inactivation of the group 1B phospholipase A [2] (Pla2glb) gene displayed lower postprandial glycemia than that observed in wild-type mice after being fed a glucose-rich meal [132].

On the other hand, high salt-fed Dahl S rats possess high renal nuclear factor-kappa B, NF-kappa B [58], which might predispose them to insulin resistance. This is because inhibition of the nuclear factor I-kappa B kinase B (IKBKB), which is a member of the NF-kappa B, with salicylates or through targeted gene disruption causes a dramatic improvement of insulin sensitivity in animal models of insulin resistance such as ob/ob mice and obese Zucker fatty rats [59,60]. It also reversed hyperglycemia, hyperinsulinemia, and dyslipidemia in obese rodents. On the other hand, activation or overexpression of the nuclear factor I-kappa B (IKBKB) attenuated insulin signaling in cultured cells [60].

As such, common variant (s) in the TNF-α, JNK, or NF-kappa B might predict a powerful inflammatory reaction in response to high salt and might possibly explain insulin resistance in Dahl S rats.

#### Factors implicated in glucose utilization by the cell

**Figure 6:**
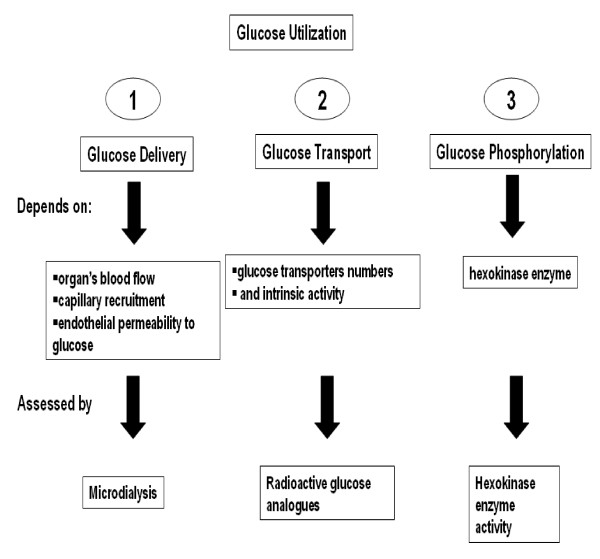
**Steps Involved in Glucose Utilization**. Schematic representation of the three steps involved in glucose utilization. In most cases, glucose transport is believed to be the rate limiting step. However, in conditions of hyperinsulinemia like that seen in high-salt-fed Dahl S rats, the rate limiting step may switch to phosphorylation [63,64]. Pertubations in any of these steps might precipitate the high salt-induced insulin resistance in Dahl S rats.

Glucose utilization requires three steps: i) delivery of glucose to the plasma membrane, which depends on the organ's blood flow, capillary recruitment and endothelial permeability to glucose [61], ii) facilitated transport across the membrane, which mainly relies on the glucose transporters numbers and intrinsic activity [61] and iii) intracellular phosphorylation to glucose-6-phosphate by a hexokinase cytosolic enzyme (HK) (Figure 6) [62].

In most cases, glucose transport is believed to be the rate limiting step. However, in conditions of hyperinsulinemia like that seen in high-salt-fed Dahl S rats, the rate limiting step may switch to phosphorylation [63,64]. Perturbations in any of these steps might precipitate the high salt-induced insulin resistance in Dahl S rats. Variations in the genes proposed in the current study might affect any of the three steps involved in glucose utilization. As for the hexokinase enzyme, increased adrenal content of norepinephrine and epinephrine in high-salt-fed Dahl S *versus *R rats might contribute to a defective hexokinase enzymatic activity in Dahl S rats [65]. Epinephrine decreases glucose clearance in vivo upon insulin stimulation [66,67] and suppresses insulin-stimulated glucose uptake in skeletal muscles [68-70]. This is possibly by: i) stimulating glycogenolysis and accumulating glucose-6-phosphate [68,71,72] (a strong inhibitor of the hexokinase enzyme) [73,74]; or ii) by reducing glucose transport across the membrane [75-77].

Despite comparable muscle and adipose tissue GLUT4 protein levels in Dahl S *versus *R rats on high and normal salt diet, Dahl S rats showed reduced glucose uptake by the muscle [19]. Reduced glucose uptake in high salt-fed-Dahl S rats might be explained by the high salt-induced release of epinephrine and norepinephrine that directly and indirectly inhibit glucose phosphorylation and utilization. Moreover, high salt causes solubilization of the hexokinase and subsequent inhibition [78], which might be an additive mechanism in reducing glucose uptake by peripheral tissues in Dahl S rats. As such, high salt combined with genetic differences in Dahl S *versus *R rats might predispose them to insulin resistance.

### Hypothesis and objectives

#### General Hypothesis

High salt diet combined with genetic differences along the insulin signaling and inflammatory pathways predict a powerful inflammatory response and susceptibility to high salt-induced insulin resistance in Dahl S rats.

#### Specific Hypotheses

○ Dahl S rats harbour common variant(s) in the genes encoding the insulin receptor substrates-1 and/or-2 and/or the glucose transporter proteins.

○ Dahl S rats will display a more powerful inflammatory response to high salt-induced oxidative stress because of common variant(s) in the genes encoding the tumor necrosis factor-alpha, c-jun terminal kinase and the nuclear factor-kappa B.

○ Dahl rats will show reduced glucose delivery, transport and phosphorylation in liver and kidney tissues compared to Dahl R rats. These differences will be related to the combined effect of high salt diet and genetic differences along the insulin signaling and inflammatory pathways.

### Objectives

I) To test whether or not Dahl S rats are more susceptible to high salt-induced insulin-resistance than a matched Dahl R rats, it is essential to assess and compare genes encoding the insulin receptor substrates -1 and -2 and genes encoding the glucose transporter proteins as follows:

a) Exhaustive candidate gene screening will be done in Dahl S *versus *R rats.

b) mRNA, protein and enzyme activities of genes involved in the insulin signaling pathway (IRS-1, -2, GLUTs) will be examined on normal, low and high salt diet.

II) To test whether or not Dahl S rats are more susceptible to high salt-induced inflammation than a matched Dahl R rats, it is essential to first assess and compare genes related to the inflammation pathway (NF-kappa B, TNF-α, JNK) and determine if they differ within or between populations as follows:

c) Exhaustive candidate gene screening will be done in Dahl S *versus *R rats.

d) mRNA, protein and enzyme activities of genes involved in the insulin signaling pathway (IRS-1, -2, GLUTs) will be examined on normal, low and high salt diet.

III) To test the combined impact of salt and variations along the insulin and inflammatory pathways, measurement of glucose delivery to the plasma membranes, glucose transport across the plasma membrane and glucose phosphorylation will be performed on normal, low and high salt diet.

IV) To test if these variations identified in objectives I and II are associated with susceptibility to high salt induced insulin resistance, haplotype analyses on candidate genes identified will be of importance.

### Methodology

Question 1: Do variations in genes encoding the insulin receptor substrates -1 and -2 and/or genes encoding the glucose transporter proteins predict salt-induced insulin resistance in Dahl S rats?

Aim 1: Determination of polymorphisms in genes encoding for IRS-1, -2 and the GLUTs proteins

#### Animals

To identify putative gender differences to susceptibility to high salt-induced insulin resistance, four-week-old male and female Dahl salt-sensitive (Dahl S) (n = 200) and salt-resistant (Dahl R) (n = 200) rats will be purchased from Harlan Sprague Dawley (Indianapolis, IN) and will be fed a standard rodent diet containing 0.3% NaCl (normal diet group) or 8% NaCl (high-salt group) or 0.03% NaCl (low-salt group) during a 4-week experimental period. The rats will be housed in a room maintained at constant humidity (60 ± 5%), temperature (23 ± 1°C), and light cycle (12 hours: 0700 to 1900). Food and tap water will be available ad libitum throughout the study. All experimental procedures will be carried out in accordance with the guidelines of the University of Ottawa Animal Care Committee for the care and use of laboratory animals. The rats will be characterized before and after normal, high and low salt diet in terms of: body weights, food intakes, blood pressures and plasma parameters (Fasting blood glucose, fasting plasma insulin). The blood pressure of the rats will be measured every other day by the tail-cuff method using a Softron BP-98A automatic sphygmomanometer [19]. Rats will be acclimated to the procedure daily during the week leading up to study. Blood glucose and plasma will be assayed using the glucose oxidase and radioimmunoassay methods, respectively. This will be performed every week using blood samples obtained from a tail vein (1 ml) [19]. Measurements of blood pressure, blood glucose and plasma insulin will continue till the fourth week post treatment. Prior to sacrificing the rats, Dahl S *versus *R rats will be confirmed as insulin resistant using the hyperinsulinemic-euglycemic clamp procedure [19].

#### Genomic DNA isolation and organ collection

Rats will be euthanized 4 weeks post treatment and genomic DNA will be isolated from white blood cells using Qiagen Human FlexiGene DNA kit according to the instructions provided by the manufacturer. 5 mls of blood/rat are sufficient to produce ample DNA for our study. Kidney and liver tissues will be collected and immediately flash frozen in liquid nitrogen for mRNA and protein extraction. Kidney and liver tissues are good candidate tissues owing to their importance in the pathogenesis of insulin resistance. Abnormal responses of insulin receptor mRNA levels to high salt intake were reported in kidneys and livers of animal models of insulin resistance such as the spontaneously hypertensive rats (SHR) and the fructose-fed rats [79,80].

#### Search for polymorphisms in genes encoding for IRS-1, -2 and glucose transporter proteins

Variants currently identified in genes encoding the IRS-1, IRS-2 and GLUTs proteins correlate with insulin resistance. In rats (Sprague Dawley), only one non synonymous polymorphism has been reported in the *IRS-1 *gene so far. However, functional studies are yet to be determined (Tables 1 and 2). Two independent methods of genotyping namely DHPLC and sequencing will be combined as was done previously [81-84]. DHPLC offers sensitivity of 95%–100% and specificity of 100% [85], while sequencing offers sensitivity of 99.7%–100% and specificity of 100% respectively [86]. Combining these highly sensitive and specific methods of genetic screening will help eliminate any false positive or false negative results that might occur with one method of genetic screening. Sequences will be retrieved from the GenBank [GenBank: *IRS-1 *NM_012969;*IRS-2*: XM_001076309]. Dahl S and R rats' sequences will be compared to each other and to the Rattus Norvegicus (Brown Norway rat) sequence readily available in the Rat Genome Database. Brown Norway rat sequence will serve as an additional control.

Aim 2: Determination of mRNA and protein expression levels for genes encoding IRS-1, -2 and glucose transporter proteinsm

#### RNA Quantification

Kidney, liver and brain tissues will be utilized for total RNA extraction using the Qiagen RNeasy Mini Kit [87-89]. Fold differences in mRNA levels for genes encoding IRS-1, -2 and the GLUTs glucose transporter proteins will be obtained using quantitative RT-PCR [90].

#### Protein Quantification

ReadyPrep™ Protein Extraction Kit (Signal), from Bio-Rad, will be utilized to extract total protein from the kidney, liver and brain tissues of Dahl rats after 4 weeks. The availability of an antiserum specific for the IRS-1 and -2 and GLUT proteins (Upstate Biotechnology Inc. Lake Placid, NY) will allow us to probe for these proteins in kidney, liver and brain cell homogenates.

Aim 3: Are IRS-1 and -2 differentially phosphorylated on serine residues

Variations in genes encoding IRS-1 or -2 might be at the level of DNA (genetic variants) or at the level of serine phosphorylation. In order to examine differential serine phosphorylation of IRS-1 and -2 in Dahl S, *versus *R rats, anti-IRS-1 phosphoserine antibody will be utilized to probe kidney, liver and brain samples from low, regular and high salt diet. Fold increase or decrease in IRS-1 and -2 serine phosphorylation in Dahl S rats post dietary treatment will be compared with control Dahl R rats.

Ogihara et al., in 2002 reported an enhanced tyrosine phosphorylation of the insulin receptor, insulin receptor substrates, PI3K activation and serine phosphorylation of Akt in Dahl S *versus *R rats [19]. However, there were some limitations to the procedures they used. First, Dahl rats were fasted for 12 hours prior to the experiment assessing IRS-1 and -2 phosphorylation. Fasting is reported to enhance insulin-induced IRS-1 and IRS-2 tyrosine phosphorylation and association with PI3-kinase in liver and muscle of animal models of insulin resistance (streptozotocin treated rats) [91]. Secondly, the authors injected insulin in the portal vein of rats once after which immediately they harvested livers and hindlimb muscles for measuring the IRS-1 and -2 levels and tyrosyl phosphorylation. Co-immunoprecipitation experiments of IRS-1 and -2 were done in triplicates. Regardless of the fact that the authors has done the insulin injection once and then repeated the immunoblotting experiment three times on the harvested liver and hindlimbs of Dahl rats, it is documented that within 1 minute of acute insulin stimulation, enhancement of the insulin signaling pathway and augmentation of IRS content and tyrosyl phosphorylation together with PI3K activation occur [92,93]. On the contrary, chronic effects of insulin have been shown to enhance IRS-1 serine phosphorylation preventing its subsequent tyrosine phosphorylation [94]. Therefore fasting and acute insulin injection in Dahl S rats (a model of insulin resistance) were additive in enhancing the insulin signaling pathway. It would have been more proper to show the impact of chronic insulin stimulation in high-salt-fed Dahl S rats without fasting or acute insulin stimulation, both of which might have misrepresented the actual defects in insulin signaling in Dahl S rats. Therefore, I propose to use the same procedure adopted by Sechi et .al., 1997, where the animals were neither fasted nor injected insulin prior to the experiment [95].

It is also possible that even in the presence of enhanced IRS-1 and/or -2 tyrosyl phosphorylation in Dahl S rats that enhanced serine phosphorylation in Dahl S rats would be the mechanism behind high salt-induced insulin resistance in this model. Serine phosphorylation might induce some conformational change that does not affect tyrosine phosphorylation but inhibits the insulin signaling pathway, via rapid tyrosyl dephosphorylation and deactivation or by acting as inhibitors to the insulin receptor kinase [40-42].

High salt might as well exert an additional inhibitory effect on *IRS-1 *and *-2*, possibly by interacting with a sodium-binding sequence [13,14] on the *IRS-1 *and/or *-2 *genes to suppress their mRNA and/or protein levels in Dahl S *versus *R rats. I was able to locate a sodium response element in the proximal promoters of IRS-1 and IRS-2 genes. As such, associations between high salt intake and insulin resistance might exist through the *IRS-1 *and/or *-2 *genes.

Aim 4: Determination of the impact of any variant/variants on the activity of IRS-1 and IRS-2, GLUTs

To test the impact of identified 5' flanking region variant(s) on the activity of IRS-1, IRS-2 and GLUTs, reporter genes can be used to assay for the activity of the promotor in a cell. The reporter gene (e.g. luciferase or GFP) is placed under the control of the *IRS-1 *or *IRS-2 or GLUT *promotor and the reporter gene product's activity is quantitatively measured. The results are normally reported relative to the activity under a "consensus" promoter known to induce strong gene expression. For variants identified in the coding region, we will assess the impact of such variant in altering the resultant amino acid sequence. The *IRS-1 *and *IRS-2 *constructs will be prepared using the same techniques we previously optimized [87,88,90]. Briefly, RT-PCR will be employed to amplify full length genes with the variant (s) identified from Dahl S rats' cDNA. Full length constructs from Dahl R rats' cDNA will be used as a control. Representative kidney and liver cell lines such as COS-7 cells and HepG2 cells respectively will be transfected with either the mutant or the corresponding wildtype as control. mRNA and protein levels will be further assessed by QRT-PCR and western blotting respectively to test the impact of such variant (s) in altering the mRNA and/or protein levels. Co-transfection studies will be performed to investigate the impact of combined variants in altering the mRNA and protein expression of IRS-1 and IRS-2, and whether a synergistic or an inhibitory response will be the outcome of such combination.

Question 2: What genes, independently or in synergy, along the inflammatory pathway are predictive of high salt-induced insulin resistance in Dahl S rats?

Aim 1: Determination of expression levels of candidate genes along the inflammatory pathway

The somewhat disappointing results of linkage studies for multi-factorial phenotypes coupled with the theory that an individual phenotype results from the cumulative effects of several contributing loci, has lead genetic investigators back to the study of candidate genes in case-control designs [96]. Identification of such candidates has become increasingly rapid with modern molecular methods such as microarrays. High-throughput microarray platforms are capable of measuring the expression of thousands of genes simultaneously allowing for the identification of genes that are differentially expressed in tissues from various individuals. Despite the recognized limitations (variation in expression resulting from polymorphism, environmental variation, or RNA quality) they can potentially reveal novel molecular mechanisms contributing to phenotypes as well as uncover alternate candidate genes derived without previous bias. As such, cDNA microarrays offers:i) detection of previously unsuspected genes with a likely role in inflammation, ii) follow-up of much over and underexpresion of gene populations compared with controls, iii) identification of phenotypically-associated specific patterns of gene expression.

Before pursuing genes along the inflammatory pathway (*TNF-α, JNK and NF-kappa B *) (Table 5), fold differences in mRNA levels will be obtained in microarray experiments and confirmed using quantitative RT-PCR. Hence haplotype analyses will only be performed on those genes whose mRNA expression patterns, protein levels or activity, significantly differ within or between the Dahl S and R population pre and post-high salt diet. Despite the high cost associated with the use of microarrays, they will enable us to identify potential inflammatory genes whose expression might be altered by diet in Dahl S *versus *R rats, and might likely influence insulin resistance in Dahl rats. Microarrays will also eliminate the most time consuming and expensive component of a future candidate gene study, and will simply direct our genetic focus to potential and differentially expressed candidate genes.

Aim 2: Searching for polymorphisms in candidate genes along the inflammatory pathway (Methodology Section-Question 1 Aim 1).

Aim 3: Determination of the impact of any variant/variants on the activity of TNF-α, JNK and NF-kappa B (Methodology Section-Question 1 Aim 4)

Aim 4: Construction of SNPs haplotypes

For haplotype construction, previously reported polymorphisms (mostly SNPs) will be identified for each candidate gene by a careful screening of available polymorphism databases. This approach is enhanced by the completion of the rat genome sequence by the Rat Genome Sequencing Project Consortium and the rapidly growing databases of rat polymorphisms, including those of The Rat SNP Consortium. Furthermore, in order to identify potential mutations and/or new polymorphisms, a systematic screening of the proximal promoters (2500 bp) will be performed. In order to exhaustively explore genetic variations, it will be essential to combine DHPLC and sequencing (ABI 310/3100 sequencers) screenings (Methodology section Question 1). DNA from rats in each population group will be used to define the haplotypes in the genes associated with differing expression post-dietary tratemnt. Haplotypes will be inferred using the PHASE software package [97]. Highly efficient genotyping techniques, depending on the selected variant, will be used to genotype the populations (Denaturing High Performance Liquid Chromatography (DHPLC), Allele Specific Oligonucleoprobe (ASO), SNaPshot™ Multiplex System).

Question 3: What are the functional consequences of gene variations on the rate of glucose delivery to the cells, the rate of glucose transport and the rate that glucose is phosphorylated within these same cells?

Aim: Determination of glucose delivery to cell membrane, glucose transport across cell membranes and glucose phosphorylation.

Measurement of interstitial glucose is relevant as it is the substrate for glucose transport. This will be performed by microdialysis as previously described [98-100]. Measurement of intracellular glucose will be predictive of the efficiency of glucose transport across cell membranes. This can be done by radioactive glucose analogues that are either not transported or not phosphorylated to calculate glucose concentration localized to each side of the membrane [101-105]. To assess perturbations in glucose phosphorylation, measuring the hexokinase enzyme activity will be performed in kidney, liver and brain tissues of Dahl rats. This will be done as previously described [106,107].

### Anticipated results

**Figure 7:**
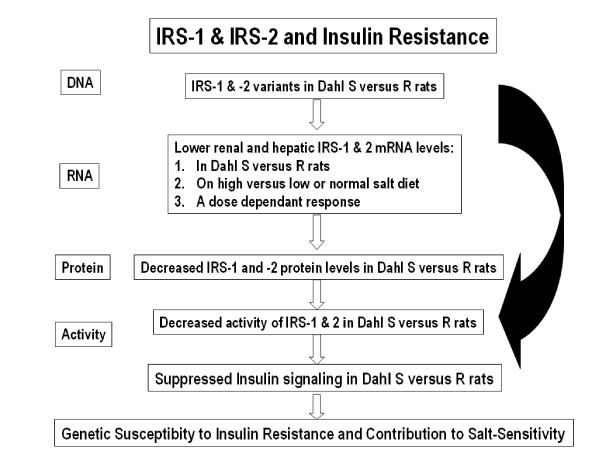
**Anticipated Results**. Summary of Expected Results in the Proposed Studies: On high salt diet, IRS-1 and/or -2 mRNA and protein levels will be diminished in Dahl S *versus *R rats. No changes are anticipated in IRS-1 and/or -2 mRNA and protein levels in Dahl S rats on normal salt diet, or in Dahl R rats on any of the dietary treatments (low, normal and high salt diet). On low salt diet, IRS-1 and/or -2 mRNA and protein levels in Dahl S rats might not change or on the contrary they might increase because of the reduced levels of sodium acting on the salt-response element in the promoter region of IRS-1 and/or -2. It is postulated in the present hypothesis that this salt-responsive element possesses an inhibitory effect on IRS-1 and/or -2. The less salt the greater the IRS-1 and/or -2 mRNA and protein levels. Serine phosphorylation is expected to be enhanced in Dahl S *versus *R rats, possibly by the higher insulin levels in Dahl S rats.

On high salt diet, IRS-1 and/or -2 mRNA and protein levels will be diminished in Dahl S *versus *R rats (figure 7). No changes are expected in IRS-1 and/or -2 mRNA and protein levels in Dahl S rats on normal salt diet, or in Dahl R rats on any of the dietary treatments (low, normal and high salt diet). On low salt diet, IRS-1 and/or -2 mRNA and protein levels in Dahl S rats might not change or on the contrary they might increase because of the reduced levels of sodium acting on the salt-response element in the promoter region of IRS-1 and/or -2. It is postulated in the current hypothesis that this sodium-binding sequence will be a salt-responsive element that will possess an inhibitory effect on IRS-1 and/or -2. The less salt the greater the IRS-1 and/or -2 mRNA and protein levels. Serine phosphorylation is expected to be enhanced in Dahl S *versus *R rats, possibly by the higher insulin levels in Dahl S rats.

**Figure 8:**
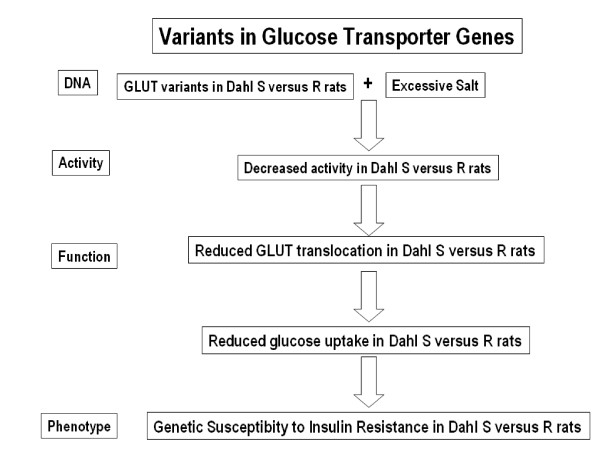
**Anticipated Results**. Summary of Expected Results in the Proposed Studies: Variants in the genes encoding the GLUT proteins in Dahl S rats, combined with high salt will decrease the activity of GLUT proteins and decrease glucose transport in Dahl S rats.

On the other hand, variants in the genes encoding the GLUT proteins in Dahl S rats, combined with high salt will decrease the activity of GLUT proteins and decrease glucose transport in Dahl S rats (figure 8).

### Perspectives

The present hypothesis together with the proposed experimental approach to test it will provide the first comprehensive evaluation of the sequence differences as well as serine phosphorylation patterns of genes along the insulin signaling pathway (*IRS-1 *and *-2 *genes and GLUTs) in Dahl S and R rats, and the first comprehensive assessment of the impact of any identified variant (s) on their activity, mRNA and protein levels in Dahl S and R rats. It will also be the first study using microarrays to identify which genes along the inflammatory pathway in Dahl S *versus *R rats show differing expression profiles pre and 4-weeks post salt overload, and if successful it will be the first to identify whether or not combined genetic differences along the insulin signaling and inflammatory pathways account for the variance in response to salt intake in or between Dahl-rat groups. Genetic factors contributing to high salt-induced insulin resistance in salt-sensitive Dahl rats will be identified, and the synergistic impact of salt diet will be described. Upon completion of the proposed research project, a sub-set of SNPs in genes encoding the *IRS-1*, *IRS-2*, glucose transporters, pro- and anti-inflammatory genes will be identified in Dahl S rats that might likely influence their susceptibility to insulin resistance and salt-sensitive hypertension.

## Competing interests

The author declares no competing interests.

## Authors' contributions

The author has conceived the hypothesis, and wrote the manuscript.
